# Adolescent Motherhood and HIV in South Africa: Examining Prevalence of Common Mental Disorder

**DOI:** 10.1007/s10461-021-03474-8

**Published:** 2021-09-27

**Authors:** Kathryn J. Roberts, Colette Smith, Lucie Cluver, Elona Toska, Siyanai Zhou, Mark Boyes, Lorraine Sherr

**Affiliations:** 1 Institute for Global Health, University College London, London, UK; 2 Department of Social Policy and Intervention, University of Oxford, Oxford, UK; 3 Department of Psychiatry and Mental Health, University of Cape Town, Cape Town, South Africa; 4 Centre for Social Science Research, University of Cape Town, Cape Town, South Africa; 5 Department of Sociology, University of Cape Town, Cape Town, South Africa; 6 Curtin enAble Institute, Faculty of Health Sciences, Curtin University, Bentley, Perth, Australia

**Keywords:** Common mental disorder, Mental health, Adolescent motherhood, HIV, South Africa, Sub-Saharan Africa

## Abstract

The mental health of adolescents (10–19 years) remains an overlooked global health issue, particularly within the context of syndemic conditions such as HIV and pregnancy. Rates of pregnancy and HIV among adolescents within South Africa are some of the highest in the world. Experiencing pregnancy and living with HIV during adolescence have both been found to be associated with poor mental health within separate explorations. Yet, examinations of mental health among adolescents living with HIV who have experienced pregnancy/parenthood remain absent from the literature. As such, there exists no evidence-based policy or programming relating to mental health for this group. These analyses aim to identify the prevalence of probable common mental disorder among adolescent mothers and, among adolescents experiencing the syndemic of motherhood and HIV. Analyses utilise data from interviews undertaken with 723 female adolescents drawn from a prospective longitudinal cohort study of adolescents living with HIV (n = 1059) and a comparison group of adolescents without HIV (n = 467) undertaken within the Eastern Cape Province, South Africa. Detailed study questionnaires included validated and study specific measures relating to HIV, adolescent motherhood, and mental health. Four self-reported measures of mental health (depressive, anxiety, posttraumatic stress, and suicidality symptomology) were used to explore the concept of likely common mental disorder and mental health comorbidities (experiencing two or more common mental disorders concurrently). Chi-square tests (Fisher’s exact test, where appropriate) and Kruskal Wallis tests were used to assess differences in sample characteristics (inclusive of mental health status) according to HIV status and motherhood status. Logistic regression models were used to explore the cross-sectional associations between combined motherhood and HIV status and, likely common mental disorder/mental health comorbidities. 70.5% of participants were living with HIV and 15.2% were mothers. 8.4% were mothers living with HIV. A tenth (10.9%) of the sample were classified as reporting a probable common mental disorder and 2.8% as experiencing likely mental health comorbidities. Three core findings emerge: (1) poor mental health was elevated among adolescent mothers compared to never pregnant adolescents (measures of likely common mental disorder, mental health comorbidities, depressive, anxiety and suicidality symptoms), (2) prevalence of probable common mental disorder was highest among mothers living with HIV (23.0%) compared to other groups (Range:8.5–12.8%; Χ^2^ = 12.54, p = 0.006) and, (3) prevalence of probable mental health comorbidities was higher among mothers, regardless of HIV status (HIV & motherhood = 8.2%, No HIV & motherhood = 8.2%, Χ^2^ = 14.5, p = 0.002). Results identify higher mental health burden among adolescent mothers compared to never-pregnant adolescents, an increased prevalence of mental health burden among adolescent mothers living with HIV compared to other groups, and an elevated prevalence of mental health comorbidities among adolescent mothers irrespective of HIV status. These findings address a critical evidence gap, highlighting the commonality of mental health burden within the context of adolescent motherhood and HIV within South Africa as well as the urgent need for support and further research to ensure effective evidence-based programming is made available for this group. Existing antenatal, postnatal, and HIV care may provide an opportunity for mental health screening, monitoring, and referral.

**Supplementary Information:**

The online version contains supplementary material available at 10.1007/s10461-021-03474-8.

## Introduction

Sub-Saharan Africa is home to the fastest growing adolescent population (10–19 years) [1] in the world, expected to reach 435 million by 2050 [2]. As such, the promotion of adolescent wellbeing—of which mental health is a core component—is critical to the success and prosperity of individuals, families, and to the region as a whole. Poor mental health within adolescence has been found to impact both physical and mental morbidity within adulthood [3–9] and, has been found to have broad societal level impacts on healthcare systems, workforce engagement and, consequently, broader socio-economic outcomes [10–12]. Despite this, mental health is an often neglected global health priority, particularly with regard to children and adolescents [9, 13–15]. Yet, 50% of mental health disorders are established before 14 years of age, and 75%, before the age of 24 years [3]. Globally, mental health disorders impact approximately 10–20% of children and adolescents [13, 16] however, there remains a dearth of prevalence estimates from low and middle income countries which may skew such global data [17–20]. Likewise, there is limited data relating to the prevention and treatment of poor mental health among children and adolescence outside of the field of developmental disability [20]. To promote the success and prosperity of adolescents within the sub-Saharan African region, an increased understanding of mental health, inclusive of when poor mental health may be compounded by other syndemic conditions (in this instance adolescent pregnancy and HIV), is necessary to inform impactful policy and programming relating to the assessment, treatment, and overall experience of mental health for adolescents.

South Africa has one of the highest rates of adolescent pregnancy (10–19 years) in the world—recent estimates suggest that 19% (95% confidence interval: 16–22%) of female adolescents have experienced pregnancy [21]. In addition to navigating a broad development period, denoted by substantial psychological, social and, biological changes, pregnant and parenting adolescents (both adolescent mothers and fathers) must also traverse pregnancy, parenting, and childrearing. Adolescent pregnancy and parenting are associated with adverse outcomes including a higher likelihood of complications during pregnancy and childbirth, adverse neonatal outcomes, repeat pregnancy, harsher parenting practices and development difficulties among their children [22, 23]. In addition, adolescent pregnancy has previously been found to be associated with poor mental health [23, 24]. Given the sensitivity of the adolescent developmental period, poor mental health during this phase may present distinctive challenges compared to pregnancy and parenthood during adulthood. Poor mental health within pregnancy and parenthood may have negative implications for maternal and child health, attachment and bonding, child development outcomes [24–28] and, at its worst, mortality—with maternal suicide linked to poor mental health. Yet, there is limited literature exploring adolescent mental health within pregnancy and parenting from the sub-Saharan African region. Here, adolescents are exposed to numerous risk factors for poor mental health including poverty, violence exposure and, high levels of communicable disease (i.e. HIV). Risk of poor mental health may therefore be further compounded by the experience of adolescent pregnancy within such settings.

The HIV epidemic within South Africa remains the largest in the world despite huge advances within the past decades. Approximately a fifth of the population (15 + years) are living with HIV [29]. Among adolescents, HIV has been found to be associated with poor mental health. A recent systematic review identified a high prevalence of mental health problems among adolescents living with HIV (ALHIV), with 24–27% of adolescents identified as experiencing psychiatric disorder and 30–50% showing behavioural or emotional difficulties [30]. Yet, the capacity for mental health treatment and care remains restricted across sub-Saharan Africa. For adolescents living with HIV, poor mental health has implications for treatment adherence [31, 32] which in turn has implications for health outcomes and onward HIV transmission.

Given that both pregnancy and living with HIV have been found to be independently associated with poor mental health among adolescents [23, 33, 34], experiencing the syndemic of both adolescent pregnancy and living with HIV may compound poor mental health experience. Such experiences may also have a bidirectional relationship with poor mental health [23, 33, 34]. Poor mental health has previously been found to be prevalent among adult populations living with HIV within pregnancy and the postpartum period [35]—however, explorations of mental health among adolescent populations are yet to be undertaken. For those adolescents living with HIV experiencing pregnancy/parenthood, there remains additional considerations such as health, stigma, medication adherence and, both postnatal and perinatal HIV transmission [36]—all of which may be impacted by mental health experience. Thus, it is important to develop an understanding of mental health within the context of adolescent parenthood and HIV to establish the impacts for both adolescents and their children.

The mental health of adolescents living with HIV who have experienced pregnancy/parenthood (both mothers and fathers) in sub-Saharan Africa and, as such, South Africa, is a neglected topic [37]. A recent systematic review exploring common mental disorder within the context of adolescent pregnancy and HIV in sub-Saharan Africa [37] identified only a single prevalence study (undertaken in Kenya) relating to depressive symptomology among a sub-sample of adolescents living with HIV who were currently pregnant (n = 14) [28]. Given the small sample size as well as the sole focus on depressive symptomology and the pregnancy period within this study [38], an understanding of common mental disorder among adolescents living with HIV who are experiencing/have experienced pregnancy remains lacking. The review identified no studies relating to risk and protective factors for mental health, and no studies relating to the interventions for poor mental health among this group [37]. These adolescents have seemingly been side-lined from mental health agendas—there exists no evidence-based policy or programming available for this group. Explicit examinations of the mental health experience of adolescents living with HIV who have experienced pregnancy/parenthood are yet to be undertaken. To build an evidence base, the first step is to establish an understanding of potential mental health need, and how this need relates to adolescent pregnancy and/or HIV status.

This study aims to determine the prevalence of likely common mental disorder among adolescents, and specifically among those who have experienced motherhood and are living with HIV in South Africa. In line with the definition of common mental disorder utilised within the recent systematic review exploring this topic area [37], likely experience of common mental disorder was defined as the presence of at least one of depressive and anxiety, posttraumatic stress, and suicidality symptomology. Globally, depression and anxiety are prominent causes of illness and disability among adolescents [39, 40]. Given the commonality of trauma experience among South African adolescents [41], the severity of suicidality symptomology (i.e. mortality) [42] and, the potential comorbidity of posttraumatic stress and suicidality with depression and general anxiety, a broad definition of likely common mental disorder (depressive, anxiety, posttraumatic stress, and suicidality symptomology) was utilised within analyses to identify need and where support for adolescents may be required.

## Methods

### Participants and Procedure

Data utilised within these analyses are drawn from a large prospective longitudinal cohort study of adolescents in the Eastern Cape province of South Africa (n = 1526). One thousand and fifty-nine adolescents living with HIV were recruited to the study utilising records from 53 public health facilities providing antiretroviral therapy to adolescents within the province. Sampling was undertaken in two stages: (1) public health facilities were identified through the national Department of Health register and, (2) all adolescents on public health facility records that had initiated treatment in the previous 3 years were approached inclusive of those disengaged from care. Adolescents were followed up utilising community tracing methods to ensure the inclusion of both those engaged and disengaged with HIV services. At baseline, 90.1% of the eligible sample identified through clinical records were interviewed. The comparison group (n = 467) were age-matched, and selected from the same environments, co-residing with or near adolescents living with HIV study participants also completed interviews. These participants self-reported that they were not living with HIV and had not initiated antiretroviral therapy and had not experienced possible opportunistic infections, nor had history of familial HIV/AIDS, thus classified as not living with HIV for the purpose of these analyses. Baseline data collection was undertaken between February 2014 and September 2015. Follow-up data was collected between November 2015 and February 2017. The cohort had a 95.3% retention rate at follow-up (n = 1454).

All adolescents and caregivers (if adolescents were < 18 years of age) provided informed consent. To protect participants confidentially interviews were conducted in a private space chosen by the participant. Given the risk of stigma relating to HIV within the communities from which data were gathered, to further protect confidentiality of participants the study was presented as a broad study focusing on youth access to health and social care services. All participants completed interviews consisting of a detailed study questionnaire consisting of validated scales and study specific questions focused on sociodemographic characteristics, health (inclusive of mental health screening measures), relationships, communities, schooling, and managements of HIV (if appropriate). Study questionnaires were developed in conjunction with adolescents (pre-piloted with a teen advisory group local to South Africa prior to data collection), as well as local, national, regional, and global organisations. Participants completed questionnaires in their language of choice (isiXhosa or English), and data was translated and back translated if appropriate.

**Fig. 1:**
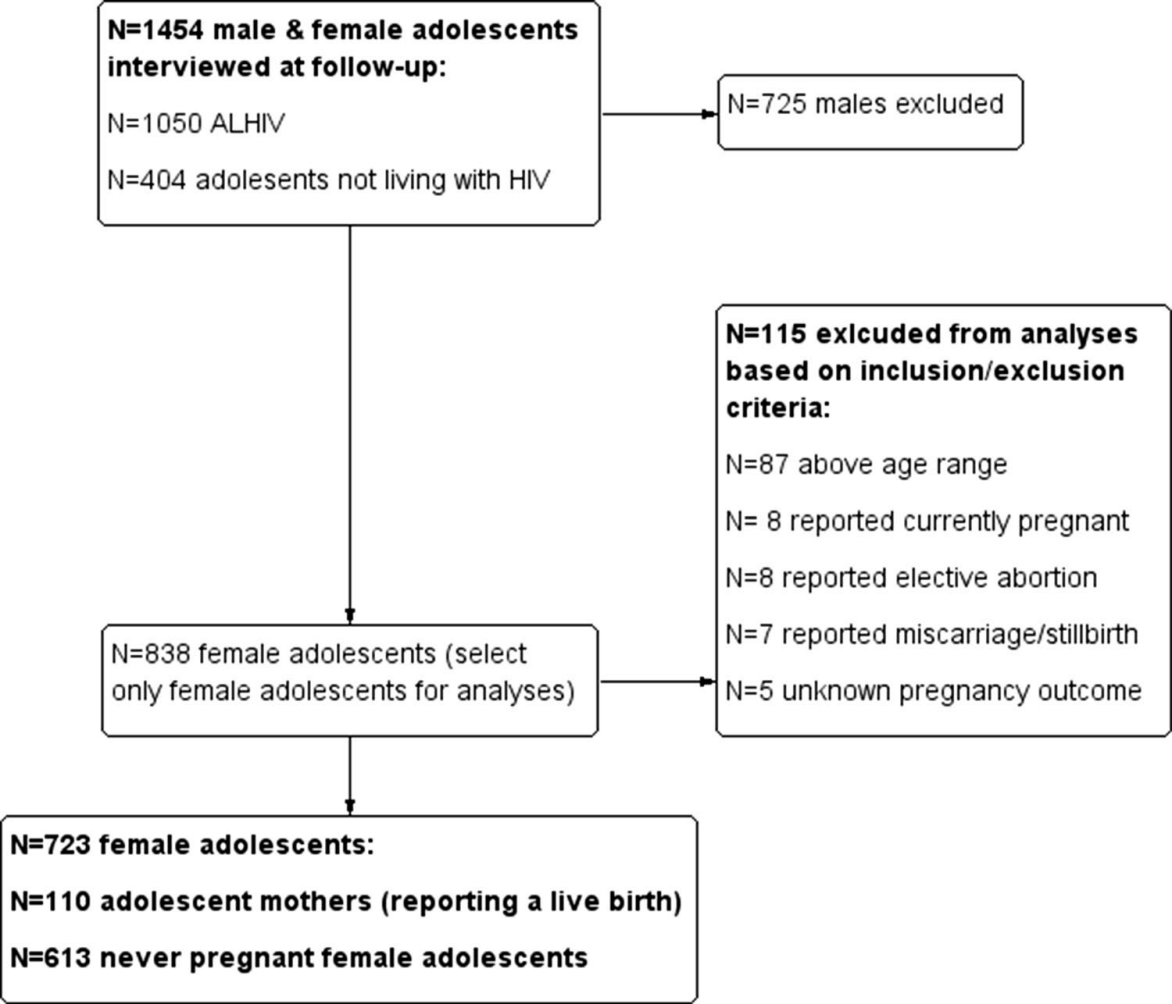
Participant selection criteria

Analyses present data relating to adolescent motherhood (previous pregnancy between 10 and 19 years of age). Only data for female adolescents’ ≤ 20 years of age were included within these analyses. Females who were 20 years of age were retained in the sample if they were within 40 weeks (average length of a full-term pregnancy) of their twentieth birthday to ensure that any reporting of pregnancy was between 10 and 19 years of age. Dates of birth and dates of interview were used to ascertain eligibility. Adolescent motherhood status was ascertained from participant self-report. Given the potentially differing mental health profiles of current adolescent mother and adolescents who had experienced elective abortion, miscarriage or still birth, adolescents who reported a pregnancy but did not report a live birth were excluded from these analyses. In total, 723 adolescents were included within analyses (see Fig. 1).

### Measures

These analyses utilise cross-sectional data. Both baseline and follow-up data were used to corroborate reported adolescent pregnancy within the sample, all other measures were obtained within the follow-up round of data collection (2015–2017; total sample n = 1454; see Fig. 1).

#### Sociodemographic Characteristics

Sociodemographic characteristics were routinely gathered during baseline data collection and corroborated during follow-up data collection. Sociodemographic characteristics included age, biological sex, dwelling location, housing, school enrolment, orphanhood status and cash grant receipt via self-report measures.

#### Motherhood Status

Adolescent motherhood (previous pregnancy and live birth between 10 and 19 years) was identified using two self-report measures [having had a previous pregnancy resulting in a live birth and having one or more biological child(ren)]. Adolescents were classified as experiencing motherhood if they scored on any one of the measures of motherhood (above). Adolescents who had experienced miscarriage, abortion, stillbirth, or the outcome of the pregnancy was unknown were excluded from analyses (see Fig. 1). Whether the pregnancy was planned and/or wanted (most recent pregnancy), age of last pregnancy (years), the number of previous pregnancies and the number of children were obtained from participant self-report.

#### HIV Status

HIV status was ascertained through clinical notes and corroborated by participant or caregiver self-report on a case-by-case basis.

#### Mental Health Status

All participants within the sample responded to all items of the four validated mental health scales used within these analyses. Cut-off scores were utilised to ascertain prevalence estimates of probable disorder.

##### Overall Mental Health Status

Two measures of overall mental health status are presented within analyses; (1) Any common mental disorder (CMD); If a participant scored above the cut-off on any of the mental health measures within the study (see below) they were classified as experiencing poor mental health. (2) Any mental health comorbidities (MHCs); If a participant scored above the cut-off on two or more of the mental health measures within the study (see below) they were classified as having mental health comorbidities.

##### Depressive Symptomology

Depressive symptomology was measured using the 10-item Child Depression Inventory short form (CDI-S) [43, 44]. Items consisted of a series of statements (i.e. ‘I like myself’, ‘I don’t like myself’ ‘I hate myself’) for which adolescents had to choose the statement that reflected how they felt. Items were scored 0–2 (0 = absent symptoms, 1 = mild symptoms and, 2 = definitive symptoms) [43, 44]. Scores of ≥ 3 (based on mild/definitive symptoms due to the small number of participants reporting definitive symptoms) [45] were used to indicate symptomology consistent with a positive screen for probable depression (binary; yes/no). The CDI-S been previously found to be highly correlated (r = 0.89) with the broader 27-item CDI scale as such scores were prorated based on the suggested inclusive cut-off within the within the full scale [43, 46, 47]. A cut-off ≥ 3 has been previously been used in high income contexts [48]. The CDI (from which the CDI-S is derived) has generally been found to have strong psychometric properties and is well validated in the sub-Saharan African region (CDI-S: α = 0.66 in the current sample) [49–52]. The CDI-S has previously been used with adolescent populations in South Africa. n adolescents [47, 53–56].

##### Anxiety Symptomology

Anxiety symptomology was measured using an abbreviated version (14 items) of the Children’s Manifest Anxiety Scale—Revised (RCMAS) [57, 58]. Adolescents identified if statements (i.e. “I worry a lot of the time”) were true for them. The 14 items were scored as “yes” (1) or “no” (0); indicative of experience consistent with anxiety symptomology and not, respectively. Scores ≥ 10 were used to indicate symptomology consistent with a positive screen for anxiety [57, 58]. The RCMAS has been validated within sub-Sharan Africa and shows good internal consistency among HIV-affected children and adolescents (α = 0.85 in the sample) [59].

##### Posttraumatic Stress Symptomology

Posttraumatic stress symptomology was measured using a 19-item version of the Child PTSD checklist [60, 61]. Adolescents were asked to think about something upsetting or frightening that has happened in their lives and identify how often they felt a series of items (i.e. “*Do you get upset when you think about what happened?*’” Items were scored 0–3 (0 “*not at all*”, 1 “*some of the time*”, 2 “*most of the time*”, 3 “*all of the time*”). Items within the checklist represent four domains relating to posttraumatic stress disorder (re-experience, avoidance, hyperarousal and, dysphoria), based on a four factor model proposed by Boyes et al. [61] for South African Adolescents [60]. Participants were classified as experiencing symptomology consistent with a probable positive screen for posttraumatic stress disorder if they scored on items across all four domains with affirmative responses (i.e. “most of the time”/”all of the time”) in the following frequencies: re-experience >  = 1, avoidance >  = 1, hyperarousal >  = 2, dysphoria >  = 2 [61]. Classifications were used to determine the presence of posttraumatic stress based on the DSM-5 criteria and were prorated based on the full Child PTSD scale [60, 62, 63]. The Child PTSD checklist showed good internal consistency within the sample (α = 0.84), has been widely used among adolescents and youth with South Africa [64, 65] and, the 19-item scale has been validated within the South African context [61].

##### Suicidality/Self Harm Symptomology

Suicidality/self-harm was measured using the five-item Mini International Neuropsychiatric Interview (MINI-KID; scored 0–5) [66]. The MINI-KID used the following questions to identify suicidal symptoms: “*In the past month did you: wish you were dead?*” “*Want to hurt yourself?*” “*Think about killing yourself?*” “*Think of a way to kill yourself?*” “*Attempt suicide?*” Participants responded “*yes*” (1) or “*no*” (0). Participants were classified as reporting suicidal symptoms if they scored on any item on the MINI-Kid [66]. Globally, the MINI-KID has been extensively validated, demonstrates good internal consistency (α = 0.89 in the current sample), and good test–retest reliability [66–68].

### Statistical Analyses

All analyses were undertaken using STATA v.15. [69] Chi-square tests (Fisher’s exact test, where appropriate) and Kruskal Wallis tests were used to explore sample characteristics (inclusive of mental health status) according to motherhood status. Prevalence and associations of likely common mental disorder with adolescent motherhood, HIV status (including the two factors combined) were described descriptively and assessed using chi-square tests. Finally, logistic regression models were used to explore the cross-sectional associations between motherhood and HIV status (inclusive of interaction effects) and common mental disorder. Interaction effects of motherhood and maternal HIV status were assessed by introducing interaction terms into the multivariable models. Adjusted odds ratios from the models including interaction terms were used to develop forest plots as a visual representation of the odds of experiencing common mental disorder among adolescent mothers who are living with HIV. Confounding factors were included in multivariable regression models if they were identified as being relevant factors within the literature of interest or found to be associated (p =  < 0.2) [70, 71] with either, or both, the predictor and outcome variables.

## Results

### Sociodemographic Characteristics

Table 1 presents sample characteristics stratified according to motherhood status. The prevalence of adolescent motherhood in the sample was 15.2%. The median age of the sample was 15 years (IQR: 13–18 years). Adolescent mothers within the sample were older compared to never pregnant adolescents [19 (IQR: 18–20) vs. 15 (IQR: 13–17) years, X^2^ = 166.9, p = 0.0001]. Over a quarter (27.1%) resided in a rural area and 15.4% lived in informal (shack) housing. Adolescent mothers were more likely to be living in informal housing comparative to never pregnant adolescents (23.5% vs. 14.1%, X^2^ = 5.73, p = 0.02). Over half of participants were orphans (52.7%). On average, the highest school grade passed was grade 9 [IQR: 7–11] and, 6.4% were not in receipt of social protection in the form of cash grants. The majority of the sample were living with HIV (70.5%). Adolescent mothers were less likely to be living with HIV compared to never pregnant adolescents within the sample (55.5% vs. 73.3%, X^2^ = 14.21, p = < 0.0001). See Table 1.

**Table 1:** Sample characteristics

	N(%)/M(IQR)			Χ^2^, p value
	Total sample (n = 723)	Adolescent mother (n = 110)	Never pregnant adolescent (n = 613)	
*Sociodemographic characteristics*				
Current age (years)	15 (13–18)	19 (18–20)	15 (13–17)	**166.86, 0.0001**
Dwelling location (rural)*	195 (27.1%)	37 (33.6%)	158 (25.9%)	2.86, 0.09
Housing (informal)*	109 (15.4%)	23 (23.5%)	86 (14.1%)	**5.73, 0.02**
Orphanhood status (one or both parents have died)	381 (52.7%)	52 (47.3%)	329 (53.7%)	1.53, 0.22
Enrolled in school	628 (86.9%)	58 (52.7%)	570 (93.0%)	**132.44, 0.0001**
Cash grant receipt	677 (93.6%)	103 (93.6%)	574 (93.6%)	0.00, 1.00
Living with HIV	510 (70.5%)	61 (55.5%)	449 (73.3%)	**14.21, < 0.0001**

Bold indicates statistical significance (*p* < 0.05)

*Missing data (total n included in analyses): Dwelling location (n = 721)|Housing (n = 709)

### Characteristics of Adolescent Motherhood

Eight respondents reported giving birth to more than one child (1.3%). The average reported age of last pregnancy within the full sample was 17 years (IQR: 16–18; Range 14–20 years). A fifth (20.9%) of adolescents reporting pregnancy reported that their last pregnancy was before the age of 16 years. Adolescents living with HIV reported a higher age at last pregnancy compared to those who were not living with HIV [17 (IQR:15–19) years vs. 17 (IQR: 15–18) years, X^2^ = 6.69, p = 0.009]. Almost all pregnancies (most recent) were unplanned or unwanted among adolescents—98.8% of pregnancies were unplanned and, 98.8% of pregnancies were unwanted.

### Prevalence of Common Mental Disorder

Table 2 presents the prevalence of probable common mental disorder for the total sample and according to the experience of adolescent motherhood. Eighty-five participants (10.9%) scored above the predetermined cut-off on at least one screening measure for mental health (depression, anxiety, posttraumatic stress, suicidality) and were classified as experiencing a probable common mental disorder. Prevalence of probable mental health comorbidities (scoring above the predetermined two or more measures for different mental health) was 2.8% in the sample. Within individual scales, 6.9% of the sample were classified as experiencing depressive symptomology, 1.4% were classified as experiencing anxiety symptomology, 0.6% reported posttraumatic stress symptomology and, 6.1% reported suicidality symptomology. Adolescent mothers reported a greater prevalence of probable common mental disorder (18.2% vs. 9.6%, X^2^ = 7.02, p = 0.008) and probable mental health comorbidities (8.2% vs. 1.8%, X^2^ = 14.15, p = < 0.0001) compared to adolescents who had never been pregnant. Within individual scales, adolescent mothers reported a greater prevalence of depressive, anxiety, and suicidality symptoms (see Table 2) than adolescents who had not experienced a pregnancy.

**Table 2:** Mental health outcomes stratified according to adolescent motherhood

Mental health outcomes	N (%)			X^2^, p value
	Total sample (n = 723) (%)	Adolescent mother (n = 110) (%)	Never pregnant adolescent (n = 613) (%)	
Any common mental disorder	79 (10.9)	20 (18.2)	59 (9.6)	**7.02, 0.008**
Any mental health comorbidities	20 (2.8)	9 (8.2)	11 (1.8)	**14.15, < 0.0001**
Depressive symptoms [scoring above cut-off (≥ 3)]	50 (6.9)	14 (12.7)	36 (5.9)	**6.80, 0.009**
Anxiety symptoms (scoring above cut-off ≥ 10)	10 (1.4)	4 (3.6)	6 (1.0)	4.83, 0.05
Posttraumatic stress symptoms (scoring above cut-off)	4 (0.6)	0 (0.0)	4 (0.7)	0.72, 1.00
Suicidality symptoms [scoring above cut-off (≥ 1)]	44 (6.1)	14 (12.7)	30 (4.9)	**10.0, 0.002**

Bold indicates statistical significance (*p* < 0.05)

Common mental disorder (scoring above the cut-off on one or more screen measure for mental health), Mental health comorbidities (experiencing two or more common mental disorders concurrently)

### Prevalence of Common Mental Disorder According to the Syndemic of Adolescent Motherhood and HIV

**Fig. 2:**
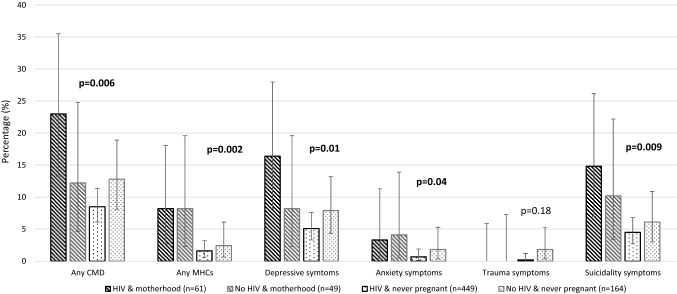
Mental health outcomes stratified accordingly to combined motherhood and HIV status. *CMD* Common mental disorder (scoring above the cut-off on one or more screen measure for mental health), *MHCs* Mental health comorbidities (experiencing two or more common mental disorders concurrently). Confidence intervals calculated at 95%. *One-sided 97.5% confidence interval

Figure 2 and Supplementary Table S1 presents the prevalence of probable common mental disorder disaggregated across four groups according to motherhood and HIV status. Prevalence of common mental disorder was found to be highest amongst adolescent mothers living with HIV (23.0%). Likewise, prevalence of depressive symptomology was found to be highest among adolescent mothers living with HIV (16.4%). Of note, the prevalence of probable mental health comorbidities was higher amongst mothers regardless of HIV status (adolescents living with HIV: 8.2%, Adolescents not living with HIV: 8.2%). Similar patterns were identified relating to anxiety and suicidality symptoms (see Fig. 2).

### Associations Between Combined Motherhood and HIV Status and Common Mental Disorder

**Fig. 3:**
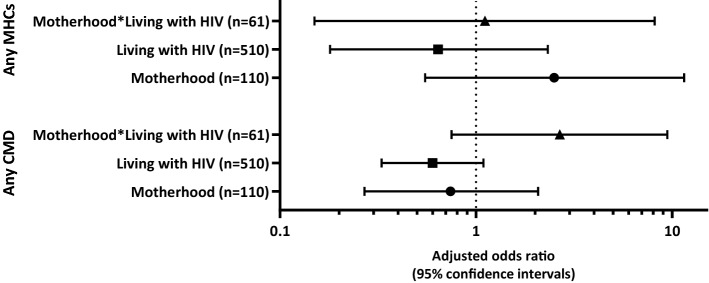
Forest plot detailing adjusted odds ratios (with 95% confidence intervals) according to combined adolescent pregnancy and HIV status. *CMD* Common mental disorder (scoring above the cut-off on one or more screen measure for mental health), *MHCs* Mental health comorbidities (experiencing two or more common mental disorders concurrently). Multivariable logistic regression models adjusted for sample characteristics [living in a rural area (yes), and living in informal housing (yes), orphan status (at least one parent has died), Enrolled in school (no), Household in receipt of cash grant (no)]

Table 3 and Fig. 3 presents logistic regression models exploring the cross-sectional associations between motherhood and HIV status and, common mental disorder. Within the multivariable models (model 1), the odds of experiencing probable mental health comorbidities were found to be elevated among those experiencing motherhood (regardless of HIV status), however, this did not reach significance (AOR: 2.64, p = 0.08; likely due to the small sample size experiencing mental health comorbidities). The association between motherhood and probable common mental disorder in the sample did not reach statistical significance (AOR: 1.30, p = 0.44). Adjusted odds ratios suggest living with HIV was protective of probable common mental disorder/mental health comorbidities, however these associations did not reach statistical significance (model 1). Within the interaction models (model 2), a trend was identified for an exacerbating interaction effect between experiencing motherhood and living with HIV, predicting an increased likelihood of probable common mental disorder (AOR: 2.67, p = 0.13). A similar trend was identified for reported depressive symptomology (AOR: 3.23, p = 0.12), identifying depressive symptomology as a likely driver of reported probable common mental disorder within these analyses.

**Table 3:** Multivariable logistic regression models exploring the association between motherhood and HIV status and, prevalence of common mental disorder

	Any common mental disorder		Any mental health comorbidity		Depressive symptoms [above cut-off (≥ 3)]		Anxiety symptoms (above cut-off ≥ 10)		Posttraumatic stress symptoms (above cut-off)		Suicidality symptoms [above cut-off (≥ 1)]	
	OR (95% CI)	p	OR (95% CI)	p	OR (95% CI)	p	OR (95% CI)	p	OR (95% CI)	p	OR (95% CI)	p
Model 1*												
Motherhood (n = 110)	1.30 (0.67–2.55)	0.44	2.64 (0.89–7.90)	0.08	1.66 (0.75–3.68)	0.21	1.79 (0.41–7.81)	0.44	1		1.59 (0.71–3.57)	0.26
Living with HIV (n = 510)	0.76 (0.45–1.29)	0.31	0.67 (0.25–1.80)	0.43	0.74 (0.39–1.42)	0.37	0.49 (0.13–1.85)	0.29	0.14 (0.009–2.09)	0.16	0.84 (0.42–1.68)	0.63
Model 2*												
Motherhood (n = 110)	0.74 (0.27–2.07)	0.57	2.50 (0.55–11.50)	0.24	0.85 (0.25–2.89)	0.80	1.27 (0.18–9.05)	0.81	1		1.36 (0.41–4.54)	0.62
Living with HIV (n = 510)	0.60 (0.33–1.09)	0.10	0.64 (0.18–2.32)	0.50	0.55 (0.27–1.14)	0.11	0.36 (0.07–2.00)	0.24	0.14 (0.009–2.09)	0.16	0.78 (0.34–1.78)	0.55
Motherhood*living with HIV (n = 61)	2.67 (0.75–9.44)	0.13	1.11 (0.15–8.14)	0.92	3.23 (0.72–14.40)	0.12	2.09 (0.14–30.98)	0.59	1		1.31 (0.29–5.93)	0.73

Common mental disorder (scoring above the cut-off on one or more screen measure for mental health), Mental health comorbidities (experiencing two or more common mental disorders concurrently)

*Model 1/Model 2: Multivariable logistic regression models adjusted for sample characteristics [living in a rural area (yes), and living in informal housing (yes), orphan status (at least one parent has died), Enrolled in school (no), Household in receipt of cash grant (no)]

## Discussion

This is the first known explicit exploration of mental health among adolescent mothers within sub-Saharan Africa with a focus on HIV status. Utilising data from a large prospective cohort study of adolescents living with HIV and a comparison group of adolescents not living with HIV within South Africa, this study explores the prevalence of probable common mental disorder according to experience of motherhood and, the syndemic of adolescent motherhood and HIV. The prevalence of adolescent motherhood was 15.2% (a figure in line with current national estimates) [21] and, over a tenth (10.9%) were classified as reporting probable common mental disorder (either depression, anxiety, posttraumatic stress, or suicidality symptomology). These prevalence data indicate a need for attention to both adolescent mental health and particularly within the context of adolescent motherhood. The detailed exploration of associations in this study reveals three core findings. First, poor mental health was higher among mothers compared to adolescents who had never experienced pregnancy. Secondly, within univariate analyses prevalence of probable common mental disorder was found to be highest among adolescent mothers living with HIV (23.0%) compared to those experiencing motherhood in the absence of a HIV diagnosis and non-mothers. Thirdly, prevalence of probable mental health comorbidities within unadjusted analyses (scoring above the cut-off on two or more measures of mental health symptomology) was found to be higher among adolescent mothers, regardless of HIV status, indicating that experience of pregnancy/motherhood may drive more complex mental health burden. This finding was supported within adjusted models, as a trend for adolescent mothers to be marginally more likely to report probable mental health comorbidities was identified. These findings highlight the commonality and potential complexity of mental health burden within the context of adolescent motherhood and HIV within South Africa and, the urgent need for further research and effective support for this group.

These findings extend literature identifying elevated prevalence of mental health burden among adolescent mothers [23, 24] and, highlight increased mental health burden (likely driven by probable depressive symptoms) among adolescents experiencing the dual impact of motherhood and living with HIV. As such, findings address a critical evidence gap as a recent systematic review identified no existing studies reporting the prevalence of common mental disorder among adolescent mothers living with HIV [37]. Adjusted models within analyses also highlight the potential complexity of probable common mental disorder among adolescent mothers within the context of HIV. While results suggest that adolescent motherhood may be associated with elevated poor mental health, adjusted models also indicate that living with HIV was potentially protective of mental health for this sample—a finding in contrast to previous literature. It should be noted that these associations did not reach statistical significance. Despite this, these findings, warrant further study to explore potential factors contributing to this pattern of results i.e. mode of HIV infection, time since HIV diagnosis, engagement with HIV services, resilience or access to social support may have implications for mental health within this population. Findings also highlight the experience of mental health comorbidities within adolescent motherhood, broadening previous investigations exploring singular mental health disorder [28]. The evidence base on mental health and pregnancy/motherhood is often limited to depressive symptomology with a reduced focus on anxiety, posttraumatic stress, and suicidal behaviours. In addition to highlighting elevated depressive symptomology among adolescents who have experienced motherhood in the sample, these data identify both anxiety and, worryingly, suicidality symptomology as prevalent among adolescent mothers. These data extend emerging literature identifying the commonality of suicidal ideation within pregnancy among HIV-affected populations in South Africa [72], providing evidence relating to adolescents. Furthermore, these data highlight the potential complexity of mental health experience (demonstrated through the presence of probable mental health comorbidities) for this group.

For adolescent mothers living with HIV, elevated common mental disorder is of particular concern as poor mental health has been found to impact HIV treatment uptake and adherence [73, 74], inclusive of PMTCT [75]—potentially having negative health consequences for both the adolescent and their child(ren). Mental health challenges have previously been found to be associated with parenting challenges i.e. harsh parenting practices [23] and sub-optimal child development outcomes [24, 28], bringing to light the importance of supporting adolescent maternal mental health for both the individual and their child(ren). A recent systematic review exploring psychosocial interventions for pregnant and parenting adolescents identified 17 interventions studies with small to moderate effects on positive mental health however, no studies were identified within low and middle income countries [76]—highlighting the need for tailored intervention among this group. There is a robust literature on effective interventions for maternal common mental disorder (mostly focused on depression) [77], inclusive of interventions for populations living with HIV within South Africa i.e. psycho-educational interventions [35, 78, 79]. Adapting such interventions for adolescents living with HIV may be of benefit. Future research may also be well placed to explore modifiable factors that contribute to common mental disorder among adolescent mothers living with HIV to better inform future programming. Possible factors driving elevated common mental disorder among this group may include stigma (related to both adolescent pregnancy/motherhood and HIV), a lack of social support, poverty, parenting stress, knowledge, and timing of HIV diagnosis and, child HIV status [35, 80–82]. For the majority, contact with health services likely increases for these adolescents during the pregnancy and post-partum period. Inherently, this contact with antenatal and postnatal care services (as well as HIV care services for adolescents living with HIV), provides an opportunity to amalgamate differing branches of health service through providing screening, monitoring, and referral for common mental disorder.

These findings should be interpreted within the context of study limitations. Firstly, the data presented are cross-sectional, as such the direction of causality cannot be established. Further, data is drawn from a sample of adolescents predominantly living with HIV (70.5%), which not representative of national HIV prevalence estimates within South Africa [83]. Hence, this may have implications for the interpretation of results, as those living with HIV are over represented, thus limiting the generalisability of findings. Nevertheless, this study addresses a critical evidence gap relating to the exploration of mental health symptomology among in adolescent mothers living with HIV and provides a foundation for future studies exploring such topics. Secondly, the data presented are mostly self-report, including motherhood status and, mental health status. This remains common practice within the field of global mental health and was deemed the best available data given the setting of data collection and the scarcity of mental health/clinical services within the locality. Mental health symptoms above a cut-off are not diagnostic but give a clear indication of potential referral need and disorder. Thirdly, while these analyses use validated cut-offs on screening measures of mental health, these data do not examine the variability within the mental health experience (i.e. variation in screening scores). In recent years, there has been a call to shift away from binary classifications of mental disorder towards a continuum approach to mental health to better reflect the diversity and complexity of mental health experience [84, 85]. However, this is yet to be implemented within the global mental health field at scale [84]. Thus, to be able to establish need in relation to mental health in these early investigations of adolescent mothers living with HIV a binary classification system was utilised within analyses. While it is important not to detract from the contextual understanding of mental health experience or pathologise adolescents through such labelling, it is essential that poor mental health symptomology is identified to ensure that support can be provided to those in need. The classifications utilised within analyses were inclusive, both within the definition of common mental disorder utilised and the cut-offs used, to ensure that potential need was identified in this sample. While all measures have previously been used in populations in South Africa, further research is still required to confirm psychometric properties in relation shortened versions of some of the scales i.e. the CDI-S and Child PTSD checklist. Fourthly, given the distribution of participants within groups established from combined motherhood and HIV status, some of the regression models undertaken as part of these analyses may have been underpowered to detect associations. Nevertheless, trends within the models were identified and, the prevalence data within these analyses contribute to a critical gap within the evidence base relating to adolescent mothers living with HIV. Fifthly, these analyses focus solely on female adolescents. It was beyond the scope of this study to explore the mental health experience of adolescent fathers. As such, there remains an absence of literature regarding the experience of who have fathered a child [37]. Finally, it was beyond the scope of this study to explore pregnancy loss, parenting of multiple children and, some of the intricacies relating to the experience of mental health burden, adolescent motherhood, and HIV [i.e. how risk and protective factors contribute to such experiences, the impact of mode of maternal HIV infection and, the timing of common mental disorder symptomology (e.g. the post-partum period)]. However, the contribution of such factors should not be excluded from the discussion regarding these experiences. Future studies should be encouraged to further examine the experience of mental health for this group to allow for a greater understanding of modifiable mechanisms of effect for common mental disorder to better inform effective interventions among this group.

## Conclusions

Mental health research, policy and provision is beginning to be given attention by international funding bodies and polygonal organisations however, resources, programming, and research capacity within the field remains low; limiting the production of evidence relevant to programming for adolescents within LMIC. For policy change to occur in relation to mental health, a core step is raising awareness of the burden of mental health challenges among the population and, identifying particularly vulnerable sub-groups to allow for targeted responses [86]. This study identified a high prevalence of probable common mental disorder among a particularly vulnerable group and provides a foundation for further exploration regarding the needs of adolescent mothers within the context of HIV. This is the first explicit exploration of probable common mental disorder prevalence among adolescent mothers living with HIV within South Africa. Three core findings emerge: (1) results highlight elevated likely common mental disorder among adolescent mothers, (2) prevalence of probable common mental disorder was found to be greatest among those adolescents experiencing the syndemic of adolescent motherhood and HIV and, (3) likely mental health comorbidities were found to be prevalent among adolescent mothers regardless of HIV status. These data focus on syndemic conditions and highlight the potential need for integrated responses for adolescent mothers within South Africa (i.e. the amalgamation of antenatal, postnatal, and HIV care, with mental health screening).

## Supplementary Information

Below is the link to the electronic supplementary material.

Supplementary file1 (DOCX 18 KB)
