# Supplementary material for: Adolescent Motherhood and HIV in South Africa: Examining Prevalence of Common Mental Disorder
Source: AIDS Behav. 2021 Sep 27;26(4):1197–210. doi: 10.1007/s10461-021-03474-8 (PMC8940800; doi:10.1007/s10461-021-03474-8)
Supplement: Supplementary file 1 — Supplementary file1 (DOCX 18 KB) [file 10461_2021_3474_MOESM1_ESM.docx]

**Supp. Table 1. Mental health outcomes stratified according to combined motherhood and HIV status**

| **Mental health outcomes** | **N (%)** | | | | | **X^2^, p-value** |
| --- | --- | --- | --- | --- | --- | --- |
|  | **Total sample (n=723)** | **No HIV & never pregnant (n=164)** | **No HIV & motherhood (n=49)** | **HIV & never pregnant(n=449)** | **HIV & motherhood (n=61)** |  |
| Any common mental disorder | 79 (10.9%) | 21 (12.8%) | 6 (12.2%) | 38 (8.5%) | 14 (23.0%) | **12.54, 0.006** |
| Any mental health comorbidities | 20 (2.8%) | 4 (2.4%) | 4 (8.2%) | 7 (1.6%) | 5 (8.2%) | **14.49, 0.002** |
| Depressive symptoms (above cut-off [≥3]) | 50 (6.9%) | 13 (7.9%) | 4 (8.2%) | 23 (5.1%) | 10 (16.4%) | **11.13, 0.01** |
| Anxiety symptoms (above cut-off ≥10) | 10 (1.4%) | 3 (1.8%) | 2 (4.1%) | 3 (0.7%) | 2 (3.3%) | **6.14, 0.04** |
| Posttraumatic stress symptoms (above cut-off) | 4 (0.6%) | 3 (1.8%) | 0 (0.0%) | 1 (0.2%) | 0 (0.0%) | 6.36, 0.18 |
| Suicidality symptoms (above cut-off [≥1]) | 44 (6.1%) | 10 (6.1%) | 5 (10.2%) | 20 (4.5%) | 9 (14.8%) | **11.56, 0.009** |

NB. Common mental disorder (scoring above the cut-off on one or more screen measure for mental health), Mental health comorbidities (experiencing two or more common mental disorders concurrently).
